# Patient satisfaction with pharmaceutical services at primary healthcare centers under the Palestinian Ministry of Health

**DOI:** 10.1186/s12913-024-10983-4

**Published:** 2024-04-24

**Authors:** Doaa Altarifi, Tahani Harb, Murad Abualhasan

**Affiliations:** 1 Ramallah & Al-Bireh Health Directorate, Ministry of Health, Ramallah, Palestine; 2 Pharmaceutical Registration Department, Ministry of Health, Ramallah, Palestine; 3 Faculty of Medicine and Health Sciences, Department of Pharmacy, An-Najah National University, Nablus, Palestine

**Keywords:** Patient satisfaction, Pharmaceutical Service, Health Care, General satisfaction

## Abstract

**Background:**

The measurement of patient satisfaction is a vital metric that enhances stakeholders to take proactive steps in improving the quality of healthcare services within medical care systems. This study assessed patient satisfaction receiving pharmaceutical services from primary health care centers in the Palestinian Ministry of Health (PMoH) governorate directorates in the West Bank.

**Methods:**

A total of 938 patients, all aged 18 years or older, completed a self-administered questionnaire. The assessment of general satisfaction was based on selected questions. Analyses were conducted to explore demographic characteristics. Mean and standard deviation (S.D.) were reported. Likert method was used to average scale satisfaction. To examine statistically significant differences, Chi-square analysis and binary logistic analysis were employed.

**Results:**

56.8% of the survey respondents were women, 57.2% were 40 years or older, and 63.2% had graduated from high school. The general satisfaction score averaged 4.10 ± 0.77 indicating good satisfaction. Patients were satisfied with interpersonal relationships, with a mean score of 4.19 ± 0.70. However, satisfaction with therapy management was lower, with a mean score of 3.99 ± 0.77 indicating moderate satisfaction. A significant factor can affect patient’s satisfaction such as the location of the pharmacy (OR = 1.720, *P* = 0.012), the waiting area (OR = 1.671, *P* = 0.002) and the cleanness of pharmacy (OR = 2.307, *P* = 0.001).

**Conclusion:**

This study underlines the main components of patient satisfaction who receive pharmaceutical services in PMoH. It is highly recommended that PMoH must address patient dissatisfaction points in a total quality management plan.

**Supplementary Information:**

The online version contains supplementary material available at 10.1186/s12913-024-10983-4.

## Background

Healthcare experiences vary among patients, and collaborative efforts by healthcare professionals aim to enhance patient satisfaction and well-being [1]. Patients expect optimal services from the healthcare system, and policymakers can influence satisfaction by improving cultural aspects, medication practices, emotional support, and staff training [2, 3]. Patient satisfaction hinges on factors such as the accessibility and quality of medical care, ease of obtaining services, effective communication with doctors, and the physicians’ expertise [4, 5]. Satisfied patients are more likely to adhere to medications and utilize healthcare services [6]. Studies indicate that improving patient satisfaction with pharmacist advice can yield financial and health benefits for both pharmacists and patients [7–9].

Demographic variations in healthcare satisfaction exist, with older and unemployed patients expressing higher satisfaction levels, and generally, female patients reporting greater satisfaction than their male counterparts [7, 10]. Recognizing these sociodemographic nuances is crucial for tailoring healthcare delivery to diverse patient groups [11].

The role of pharmacists has evolved, expanding beyond traditional dispensing to active involvement in healthcare activities. However, mismatches between patient expectations and the evolving pharmacist role can impact overall satisfaction. Patient satisfaction in pharmaceutical care involves dimensions like pharmacy performance, patient expectations, and preferences [12].

Measurement of patient satisfaction in the pharmaceutical context employs diverse instruments, focusing on service delivery, financial aspects, and overall accessibility. This multipronged approach allows for a comprehensive assessment, driving quality enhancements [13, 14]. Global healthcare strategies, exemplified by Saudi Arabia’s pharmacy planning, underscore the intrinsic value of patient satisfaction [15, 16].

Effective communication and timeliness are crucial facets shaping patient satisfaction, with waiting times for prescriptions and clear medication information being key determinants [17]. Despite understanding patient satisfaction globally, certain regions, like Palestine, lack specific exploration in this context [18–20]. Context-specific research is vital for uncovering unique dynamics shaping satisfaction within distinct healthcare landscapes.

Patient satisfaction surveys for pharmaceutical services within the Palestinian Ministry of Health (MoH) are to provide valuable insights into the quality and effectiveness of healthcare delivery, helping the MoH identify areas for improvement and allocate resources efficiently. Understanding patient perspectives and experiences, the MoH can tailor services to better meet the needs of the Palestinian patients and enhance healthcare outcomes. Moreover, monitoring patient satisfaction ensures accountability and transparency within the healthcare system, fostering trust between patients and healthcare providers. This study’s primary aim was to assess patients receiving pharmaceutical services from MoH) in the primary health care centers of the Palestinian ministry of health (PMoH) in different districts including Ramallah, Nablus, and Hebron, evaluating satisfaction levels and the factors associated with these satisfactions to identify areas for improvement. The research aimed to enhance service quality, ultimately striving for the highest possible level of patient satisfaction.

## Methods

### Inclusion and exclusion criteria

A randomly chosen sample of 938 participants, all of who were at least 18 years old and had indicated that they were prepared to take part in the study and had given their oral consent to be interviewed were recruited for the study. In the course of doing this research, essential data pertaining to demographics and socioeconomics were gathered, and the participants’ levels of satisfaction with the pharmaceutical services they received were assessed.

Participants who were under the age of 18 or who suffered from mental health conditions and received pharmaceutical treatments from the health directorates in Ramallah & Al-Bireh, Nablus, or Hebron were excluded from the study.

### Study design and sampling

A cross-sectional investigation was executed to evaluate the satisfaction of patients concerning pharmaceutical services within governmental healthcare centers under the jurisdiction of PMoH governorate directorates in the West Bank. This study was conducted between March and December 2021. In Ramallah, we opted for the Ramallah and Al-Bireh directorate to represent the central region within the West Bank. In Nablus, we selected three central clinics: Alwsta, Almakhfeia, and Balata, and similarly, three clinics were chosen in Hebron: Karantina, Alsalam, and Alrama.

These three governorate directorates were deliberately chosen to render the findings representative of the entire West Bank. Nablus health governorate directorate stood for the northern region, Ramallah & Al-Bireh health governorate directorate for the central area, and Hebron health governorate directorate for the southern part of the West Bank. The pharmacy services offered at these healthcare centers vary from those of community pharmacies, as they exclusively provide medication free of charge to patients with insurance from the Palestinian Authority. The dispensed medications are limited to those listed in the essential drug list approved by MoH. The minimal sample size was defined as 1,090 patients (350 + 360 + 380) for Ramallah, Nablus, and Hebron, respectively. To account for incomplete surveys, the sample size was increased by 10%, leading to a required sample size of 1,200 patients. The determination of the sample size relied on inputs from the registration division affiliated with Ramallah & Al-Bireh health governorate directorate, Nablus, and Hebron health governorate directorates, as well as the sample size calculator within the Raosoft Inc. program [21]. The total patient counts were 5,000, 6,000, and 12,000 for pharmaceutical services recipients in Ramallah, Nablus, and Hebron, respectively. A confidence level of 95% and a margin of error set at 5% were utilized.

### Instrument

The questionnaire was disseminated among adult patients who were 18 years of age or older. The questionnaire to assess patient satisfaction with pharmaceutical care was similar with slight modification from published literature which was written in Spanish language was then translated into English and then further translated into Arabic [22]. The survey was designed to take approximately 5–10 min to complete. To comply with the study objectives, adaptations and additions were made to the questionnaire based on content from other sources [15, 23, 24]. Participants were assured that their responses were entirely voluntary and would remain confidential (See Supplementary data). Initially, a pilot test was conducted with a group of 30 patients, and the outcomes from this phase were utilized to refine the questionnaire. Adjustments were made, which encompassed rephrasing or omitting items, shortening the length, and making modifications.

Patients filled out a self-administered questionnaire independently, with the researcher providing assistance and conducting a double-check to ensure that all questions were completed. The questionnaire comprised a total of 33 questions and was organized into two sections. The primary section focused on obtaining vital demographic and socioeconomic information through a succinct set of questions. This covered aspects like age, gender, marital status, educational attainment, employment status, and place of residence. While the secondary section gauged patient satisfaction with pharmaceutical services. This entailed evaluating the pharmacist’s effectiveness in explaining medication usage, potential side effects, time allocated for patient interaction, gathering patient-specific details, identifying medication-related issues, resolving said issues, tracking outcomes, the waiting area ambiance, service swiftness, medication availability, overall satisfaction, as well as the convenience of pharmacy location, medication quantity adequacy, comfort of the waiting area, pharmacy cleanliness, medication availability, and packaging quality.

Participants were given the choice to participate or not in the study. An informed consent was obtained from each participant before their involvement.

The Ministry of Health (MoH) granted approval for the research to be conducted in their governorate directorates in the West Bank (Ramallah, Nablus, and Hebron). Permission was obtained from each pharmacy director in the clinics to administer questionnaires to patients.

Upon completing the study, appropriate reports were compiled. These reports were sent to the Ministry of Health and the General Administration of Pharmacy. The purpose was to provide these authorities with insights and data from the study, which could guide them in taking appropriate actions to improve Palestinian pharmaceutical services.

### Statistical analysis

The results were presented in percentages (%), and the average (mean), along with the standard deviation (SD), was provided. Additionally, certain outcomes were expressed as odds ratios (OR) along with their corresponding confidence intervals (CI). The statistical analysis was performed using SPSS software version 22. Cronbach’s alpha was used to assess the internal consistency reliability of the questionnaire items. This indicates how well the items in the questionnaire correlate with each other.

Frequencies were calculated for demographic characteristics to understand the distribution of the sample. A five-choice assessment scale (ranging from ‘excellent’ to ‘poor’) was used to assess patient satisfaction. Assigned scores were: ‘excellent’ (5),' good’ (4), ‘moderate’ (3), ‘fair’ (2), ‘poor’ (1). Likert method was used to aggregate scores within each assessment scale. Mean and standard deviation (SD) were calculated for each scale to assess patient satisfaction. The scales were recoded into two categorical variables: ‘low to moderate satisfaction’ and ‘high satisfaction’. A cutoff point of 4 was used; patients with a score of 4 or less were considered to have low to moderate satisfaction, while those with a score above 4 were considered to have high satisfaction.

Bivariate analysis was conducted using the Pearson Chi-Square test to identify significant relationships between demographic, socioeconomic factors, place, supply variables, and the dependent variable (patient satisfaction).

Pearson Chi-square and *p*-values were used to determine the strength and significance of these relationships. Multivariate analysis, specifically binary logistic regression, was conducted to identify potential confounding variables that might influence the relationship between the independent variables (demographic, socioeconomic factors, place, supply variables) and the dependent variable (patient satisfaction).

## Results

Among the 1200 patients invited to complete the questionnaire, 78% of them, totaling 938 individuals, agreed to take part. These participants had availed themselves of pharmaceutical services at Ministry of Health (MoH) clinics located in the West Bank, specifically in Ramallah, Nablus, and Hebron. Within this group, 56.8% were female, 57.2% were aged 40 or older, 38.1% resided in Ramallah, 37.6% in Nablus, and 24.3% in Hebron. Moreover, 63.2% had attained a high school education or higher, 73.3% were married, and 50.5% were currently unemployed. The detailed results are shown in Table 1.

**Table 1:** Demographic data of interviewed patients who are receiving services at the MoH clinics (*N* = 938)

Demographic Variable		Statistical summary N (%)
Sex	Women	533 (56.8%)
	Men	405 (43.2%)
Age	Mean (SD)	43 (14.9)
Age category	39 years or less	401 (42.8%)
	40 and above	537 (57.2%)
Governorate	Ramallah	357 (38.1%)
	Nablus	353 (37.6%)
	Hebron	228 (24.3%)
Education	Uneducated	72 (7.7%)
	Primary school level	273 (29.1)
	High school or above	693 (63.2%)
Marital status	Single	154 (16.4%)
	Married	688 (73.3%)
	Widow / Divorced	96 (10.2%)
Employment status	Unemployed	474 (50.5%)
	Employed	464 (49.5%)

The results indicate that a significant majority, approximately 79.3%, expressed agreement regarding the convenience and comfort of the pharmacy’s location. Additionally, around 56.9% of respondents agreed that the quantity of medication provided was sufficient, while 60.1% found the waiting area to be comfortable. A substantial 86.7% of participants agreed that the pharmacy area was clean and acceptable. On the other hand, 59.2% disagreed with the statement that all required medications were readily available in the pharmacy. Finally, a noteworthy 81.1% of interviewees agreed that all the medications they received were well-packaged.

The analysis of the interview responses reveals a notable level of satisfaction, with mean scores ranging from 3.86 to 4.42 for each item related to pharmaceutical services. These scores reflect a range of moderate to high levels of satisfaction across all aspects of pharmaceutical services. Detailed results can be found in Table 2.

**Table 2:** Patients’ average satisfaction ratings for the services provided by pharmacists (*N* = 938)

No.	Item	Mean	SD
**Interpersonal Relationship**			
1	The pharmacist’s interest in your health	4.35	0.83
2	The pharmacist’s professional relationship with patient	4.22	0.86
3	The courtesy and respect shown to you by the pharmacy staff	4.42	0.78
4	The advice you get from the pharmacist about problems that might occur with your medication	4.08	0.96
5	The help you received from the pharmacist to avoid unnecessary costs related to your prescriptions	4.02	1.04
6	The amount of time the pharmacist spends with you	3.86	1.02
7	The pharmacist’s instructions on how to take your medication	4.20	0.93
8	The professionalism of all the pharmacy staff	4.30	0.82
9	The way the pharmacist answers your questions	4.27	0.88
**Managing therapy**			
10	The availability of the pharmacist to answer your questions	4.21	0.86
11	The way the pharmacist helps you in managing your medications	4.11	0.86
12	How frequently the pharmacist checks in with you about how well your medications are working	3.89	1.06
13	The pharmacist’s efforts in helping you improve your health	3.92	1.01
14	The information the pharmacist gives you about the proper storage of your medication	4.01	1.00
15	The help you get from your pharmacist when you have a health problem related to your medication	4.02	0.94
16	The written information the pharmacist provides to you about drug therapy and/or diseases	4.04	0.96
17	The information the pharmacist gives you about the results you can expect from your drug therapy	3.89	1.02
18	The pharmacist’s help when a medication doesn’t have the expected effect	3.86	0.99
19	How the pharmacist uses information about previous conditions/drugs when assessing drug therapy	3.94	0.99
20	The help received from the pharmacy staff with the administrative arrangements necessary to get therapy	4.01	0.93
21	The way your pharmacist works together with you to plan what should be done to achieve good results from your medications	3.96	0.98
22	The way your pharmacist works together with your doctor to make sure your medications are the best for you.	3.97	1.01
23	The responsibility that the pharmacist assumes for your drug therapy	4.00	0.96
**General Satisfaction**			
24	The privacy of conversations with the pharmacist	3.86	1.09
25	The amount of time it takes to get a prescription filled at your pharmacy		
26	The professional appearance of the pharmacy	4.39	0.79
27	Pharmacy’s services overall	4.21	0.90

In this research, the internal consistency reliability indices for the reliability, managing therapy, and interpersonal relationship scales were assessed using the Cronbach’s alpha test. The items measuring these factors were chosen from the questions listed in Table 3. The outcomes demonstrated the reliability and internal consistency of the results, with reliability coefficients of 0.780 for managing therapy, 0.810 for interpersonal relationships, and 0.874 for general satisfaction.

**Table 3:** Results of the internal consistency of Cronbach’s alpha test

	Item	Factors	Mean	SD	Cronbach’s alpha	NO. of item
1	Managing Therapy	10, 11, 12, 13, 14, 15, 16, 17, 18, 19, 20, 21, 22, 23	3.99	0.77	0.780	14
2	Interpersonal Relationship	1, 2, 3, 4, 5, 6, 7, 8, 9	4.19	0.70	0.810	9
3	General Satisfaction	24, 25, 26, 27	4.10	0.77	0.874	4

In this study, Chi-square tests revealed a significant difference between overall satisfaction and several categorical variables. The examined categorical variables included the convenience and comfort of the pharmacy’s location (*p* < 0.001), the appropriate quantity of medication (*p* < 0.001), the comfort of the waiting area (*p* < 0.001), the cleanliness and acceptability of the pharmacy area (*p* < 0.001), the availability of all prescribed medications in the pharmacy (*p* < 0.001), and the quality of packaging for all received medications (*p* < 0.001). The detailed results are illustrated in Table 4.

**Table 4:** Pearson Chi-square results of demographic and socioeconomic variables related to dependent variable

Variable Name		Patients’ General Satisfaction		Pearson Test	
		Low to Moderate N (%)	High N (%)	Chi-square Test	*P* Value
Sex	Men	183 (45.2%)	222 (54.8%)	0.850	0.357
	Women	257 (48.2%)	276 (51.8%)		
Age	39 and less	189 (47.1%)	212 (52.9%)	0.014	0.905
	40 and above	251 (46.7%)	286 (53.3%)		
Governorate	Ramallah	161 (45.1%)	196 (54.9%)	1.349	0.509
	Nablus	165 (46.7%)	188 (53.3%)		
	Hebron	114 (50%)	114 (50%)		
Education	Uneducated	34 (47.2%)	38 (52.8%)	1.052	0.591
	Primary school level	121 (44.3%)	152 (55.7%)		
	High school or above	285 (48.1%)	308 (51.9%)		
Marital status	Single	77 (50%)	77 (50%)	2.122	0.346
	Married	324 (47.1%)	364 (52.9%)		
	Widow / Divorced	39 (40.6%)	57 (59.4%)		
Work	Employed	221 (47.6%)	243 (52.4%)	0.192	0.662
	Unemployed	219 (46.2%)	255 (53.8%)		
The location of the pharmacy was convenient and comfortable	Agree	298 (40.1%)	446 (69.9%)	67.86	< 0.001
	Disagree	142 (73.2%)	52 (26.8%)		
Medication quantity was sufficient	Agree	201 (37.6%)	333 (62.4%)	42.76	< 0.001
	Disagree	239 (59.2%)	165 (40.8%)		
The waiting area was comfortable	Agree	203 (36%)	361 (64%)	67.67	< 0.001
	Disagree	237 (63.4%)	137 (36.6%)		
The pharmacy area was clean and acceptable	Agree	342 (42.1%)	471 (57.9%)	57.43	< 0.001
	Disagree	98 (78.4%)	27 (21.6%)		
All medications were available in the pharmacy	Agree	132 (34.5%)	251 (65.5%)	40.24	< 0.001
	Disagree	309 (55.5%)	247 (44.5%)		
All medications I received were well packaged	Agree	315 (41.4%)	446 (58.6%)	50.38	< 0.001
	Disagree	125 (71%)	52 (29%)		

The results of the binary logistic regression analysis revealed significant associations. Specifically, individuals who agreed that the pharmacy’s location was convenient and comfortable were 1.720 times more likely to have high general satisfaction compared to those who disagreed (OR = 1.720, *P* = 0.012). Similarly, those who found the waiting area comfortable were 1.671 times more likely to report high general satisfaction than those who disagreed (OR = 1.671, *P* = 0.002). Furthermore, participants who agreed that the pharmacy area was clean and acceptable were 2.307 times more likely to express high general satisfaction compared to those who disagreed (OR = 2.307, *P* = 0.001). Lastly, individuals who agreed that all medications received were well packaged were 1.655 times more likely to indicate high general satisfaction compared to those who disagreed (OR = 1.655, *P* = 0.017). Additional details, including the 95% confidence intervals, can be found in Table 5.

**Table 5:** Binary logistic regression result of the studied samples (*N* = 938)

Independent Variables	Categories	B	OR	95% C.I.		*P*-value
				Lower	Upper	
The location of the pharmacy is convenient and comfortable	Agree	0.543	1.720	1.126	2.629	0.012
	Disagree (Ref.)	-	1	-	-	-
Medication quantity was sufficient	Agree	0.237	1.267	0.904	1.776	0.169
	Disagree (Ref.)	-	1	-	-	-
The waiting area was comfortable	Agree	0.513	1.671	1.200	2.326	0.002
	Disagree (Ref.)	-	1	-	-	-
The pharmacy area was clean and acceptable	Agree	0.836	2.307	1.378	3.862	0.001
	Disagree (Ref.)	-	1	-	-	-
All medications were available in the pharmacy	Agree	0.224	1.251	0.887	1.764	0.202
	Disagree (Ref.)	-	1	-	-	-
All medications I received were well packaged	Agree	0.504	1.655	1.094	2.503	0.017
	Disagree (Ref.)	-	1	-	-	-

## Discussion

In health care services, patient satisfaction is considered an important measure that helps the stakeholder to improve the quality of the health services in medical care systems [25].

In Palestine, there are 441 primary health care centers affiliated with the Ministry of Health (MoH), distributed across 11 districts. These centers reported a total of 2,339,066 visits [26]. A questionnaire to assess patient satisfaction with pharmaceutical care in Spain was accepted by 81% of the approached patients and this acceptance rate was similar our study, where 78% of the sample patients agreed to answer the questionnaire [15]. Patients required 5 to 10 min to fill out the questionnaire, aligning with the timeframe observed in another study [27].

In this study, there were no statistically significant differences between patients based on sociodemographic characteristics (age, sex, education, marital status, employment status) (*P* > 0.05); our result aligned with other literature [28, 29]. However, some sociodemographic variables were significant in another studies where it showed that women were less satisfied than men [30], while other studies showed a higher level of satisfaction among female patients and assumed that women might be more willing to ask about their medications from the pharmacist [31]. This study also Older patients were more satisfied than younger patients. This may be due to the older age groups having fewer expectations in relation to services than younger age groups [32]. Moreover, this study revealed that patients with at least a secondary school education experienced low to moderate satisfaction compared to others. This inverse relationship between satisfaction and education was consistent with studies that found that less educated patients were generally more satisfied as they may appreciate receiving any kind of pharmaceutical care. In contrast, higher educated patients had higher expectations of the pharmaceutical services [33].

Numerous studies have demonstrated differences in satisfaction levels based on various sociodemographic factors. For instance, individuals who were divorced exhibited higher satisfaction with pharmaceutical care compared to their married counterparts, as indicated in prior research [34]. Additionally, a separate study identified a notable association between patient satisfaction and employment status, revealing that unemployed patients reported greater satisfaction with pharmaceutical care than their employed counterparts [35].

The interpersonal relationship metrics had a mean score of 4.19 and high patient satisfaction with a mean score of more than 4. This was similar to the study conducted in Iowa, U.S., that indicated that pharmacists working in these centers took good care of patients and have an excellent degree of knowledge. The amount of time the pharmacist spends with patients question had low to moderate satisfaction, similar to the study from Iowa, U.S [36]. This low satisfaction level can result from a large flux of patients visiting clinics, making the time a patient spends with the pharmacist insufficient [37].

The managing therapy metric showed high patient satisfaction with a mean score of more than 4. This is similar to the Iowa study that showed high patient satisfaction with the availability of the pharmacist to answer patient questions and the information provided by the pharmacist about health implications regarding medications [36]. Also, the literature supported that managing therapy and its related aspects, including pharmacist availability, thorough pharmacist explanations and clear labelling of drugs, pharmacist politeness, and prompt services, are strong determinants of patient satisfaction [38]. Improving pharmaceutical services can reduce the number of adverse drug reactions and thus improve overall health [39]. Another study determined that the relationship between pharmacist practices and patient satisfaction depended on the attitude of the pharmacists through different activities, such as when providing information and explanations on medication [40].

On the other hand, our study has found that patients had low to moderate levels of satisfaction to items related information provided by the pharmacist. This corresponded to a study conducted in Saudi Arabia which found that 61.2% of patients stated they did not understand the information provided by the pharmacist regarding the results the patient can expect from their drug therapy [7]. As such, managing therapy and interpersonal relationship are essential components of pharmaceutical care with a high impact on patients’ health and quality of life [22]. In a study conducted in Saudi Arabia found that 65.9% of the patients agree that pharmacists have a significant role in disease recovery and that Saudi patients show better satisfaction, perception, and appreciation of the pharmacists’ role in the health care team [41].

Patients in our study were least satisfied with the time spent waiting for the prescription to be filled. This was different from the study conducted in Brazil that showed that patients reported higher satisfaction with waiting time to obtain their medication [42, 43]. The literature showed that dispensing time is one of the dispensing indicators in Good Pharmacy Practice (GPP), which aims to provide an easy and reliable way to improve the use of medicines [44]. The reason for low to moderate satisfaction may be due to the study was conducted in public clinics that provided services to many patients.

In this study, a significant variation (*P* < 0.001) was observed between general satisfaction and the following variables: the convenience of pharmacy location, sufficient medication quantity, comfortable waiting area, the cleanliness of the pharmacy, medication availability, and well-packaged medication. This was compatible with another study conducted in Philadelphia County in Pennsylvania regarding pharmacy location showed patients were more satisfied when the pharmacy location was convenient [42]. Another study showed that comfortable facilities where the building was impressive, the waiting area/room was comfortable, and the pharmacy was sanitary were critical in the evaluation of patient satisfaction, whereas the convenience of location did not influence patient satisfaction [35]. On the other hand, our results indicate that 59.2% of the patients disagreed that all medications were available in the pharmacy. This needs further investigation in the future because of the lack of medicines due to economic, political, and financial reasons in Palestine that prevent the sufficient supply of medicines to the Ministry of Health on time. This was different from a study conducted in Brazil regarding the availability of medications, where 65.1% of patients showed they did not have problems the last time they obtained their medicines from primary health care in different cities [32].

This is the first study conducted in PMoH–West Bank to assess patient satisfaction. Nonetheless, the study has certain limitations. These include the potential for information bias arising from inaccuracies in questionnaire responses, the omission of certain parameters in the questions, which, if included, might have influenced satisfaction outcomes, and the potential impact on measuring the variables of interest.

Since this study used convenience sampling stratified by regions of eligible patients from Ramallah & Al-Bireh, Nablus and Hebron health governorate directorates, the study’s results cannot be generalized globally. The findings of this study offer insights into the universal challenge of ensuring quality healthcare delivery and patient-centered services in resource-constrained settings worldwide. As healthcare systems globally strive to enhance access, efficiency, and patient outcomes, understanding the factors influencing patient satisfaction with pharmaceutical services becomes paramount. The experiences and perceptions of patients in Palestine can resonate with individuals accessing primary healthcare services in diverse geographic, socioeconomic, and cultural contexts. By examining the drivers of patient satisfaction in this unique setting, this research contributes to the broader discourse on improving healthcare quality and patient experiences internationally, guiding policymakers, healthcare practitioners, and researchers in developing strategies to address common challenges and promote patient-centered care globally.

## Conclusion

This study emphasizes the key factors contributing to patient satisfaction among those who utilize pharmaceutical services within the (PMoH). While our survey and analyses indicated that patients in the (PMoH) directorates in the West Bank generally reported satisfaction ranging from moderate to high in interpersonal relationships and the management of therapy areas, there were specific service aspects related to therapy management that received lower to moderate satisfaction scores. These aspects include how frequently the pharmacist checks in on the effectiveness of medications, the pharmacist’s efforts in enhancing the patient’s health, and the information provided by the pharmacist regarding expected outcomes from drug therapy. Therefore, it is imperative that we incorporate strategies to address areas of patient dissatisfaction as part of a comprehensive quality management plan. We recommend enhancing pharmacists’ awareness of their strengths and weaknesses to enhance patient satisfaction. This approach involves identifying potential interventions and system improvements, as well as devising a forward-looking plan to rectify these weaknesses.

### Electronic supplementary material

Below is the link to the electronic supplementary material.

Supplementary Material 1

## Data Availability

All data generated or analyzed during this study are included in this published article.

## Abbreviations

(PMoH): primary health care centers in the Palestinian Ministry of Health
OR: Odds Ratio
SD: Standard Deviation
CI: Confidence interval
MoH: MoH
%: Percentage
N: Number
GPP: Good Pharmacy Practice
