# Supplementary material for: Patient satisfaction with pharmaceutical services at primary healthcare centers under the Palestinian Ministry of Health
Source: BMC Health Serv Res. 2024 Apr 24;24:514. doi: 10.1186/s12913-024-10983-4 (PMC11044550; doi:10.1186/s12913-024-10983-4)
Supplement: Supplementary file 1 — Supplementary Material 1 [file 12913_2024_10983_MOESM1_ESM.docx]

**Supplementary Material**

**Patient Satisfaction with Pharmaceutical Services at Primary Healthcare Centers within the Palestinian Ministry of Health**

*Doaa Altarifi^1*^, Tahani Harb^2^, and Murad Abualhasan^3*^*

^1^ Ramallah & Al-bireh Health Directorate, Ministry of Health, Ramallah, Palestine

^2^ Pharmaceutical Registration Department, Ministry of Health, Ramallah, Palestine

^3^Faculty of Medicine and Health Sciences, Department of Pharmacy, An-Najah National University, Nablus, Palestine

**Questionnaire:**

**This section is for patient demographic information.**

*Gender

Male Female

* Age in years -------------

* Education level:

No education high school/ secondary education Diploma University education and more

*Marital status

Single married divorced / widowed

* How many times did you visit the pharmacy in primary health care centers

The first time in a month more than once a month

Medication usage:

When needed or when necessary Daily Weekly

*Address

*Job

| **Interpersonal Relationship** | | | | | |  |
| --- | --- | --- | --- | --- | --- | --- |
| **No.** | **QUESTION** | **Excellent** | **Very good** | **Good** | **Fair** | **Poor** |
| 1 | The pharmacist’s interest in your health |  |  |  |  |  |
| 2 | The pharmacist’s professional relationship with you |  |  |  |  |  |
| 3 | The courtesy and respect shown to you by the pharmacy staff |  |  |  |  |  |
| 4 | The advice you get from the pharmacist about problems that might occur with your medication |  |  |  |  |  |
| 5 | The help you received from the pharmacist to avoid unnecessary costs related to your prescriptions |  |  |  |  |  |
| 6 | The amount of time the pharmacist spends with you |  |  |  |  |  |
| 7 | The pharmacist’s instructions on how to take your medication |  |  |  |  |  |
| 8 | The professionalism of all the pharmacy staff |  |  |  |  |  |
| 9 | The way the pharmacist answers your questions |  |  |  |  |  |
| **Managing therapy** | | | | | |  |
| **No.** | **QUESTION** | **Excellent** | **Very good** | **Good** | **Fair** | **Poor** |
| 10 | The availability of the pharmacist to answer your questions |  |  |  |  |  |
| 11 | The way the pharmacist helps you in managing your medications |  |  |  |  |  |
| 12 | How frequently the pharmacist checks in with you about how well your medications are working |  |  |  |  |  |
| 13 | The pharmacist’s efforts in helping you improve your health |  |  |  |  |  |
| 14 | The information the pharmacist gives you about the proper storage of your medication |  |  |  |  |  |
| 15 | The help you get from your pharmacist when you have a health problem related to your medication |  |  |  |  |  |
| 16 | The written information the pharmacist provides to you about drug therapy and/or diseases |  |  |  |  |  |
| 17 | The information the pharmacist gives you about the results you can expect from your drug therapy |  |  |  |  |  |
| 18 | The pharmacist’s help when a medication doesn’t have the expected effect |  |  |  |  |  |
| 19 | How the pharmacist uses information about previous conditions/drugs when assessing drug therapy |  |  |  |  |  |
| 20 | The help received from the pharmacy staff with the administrative arrangements necessary to get therapy |  |  |  |  |  |
| 21 | The way your pharmacist works together with you to plan what should be done to achieve good results from your medications |  |  |  |  |  |
| 22 | The way your pharmacist works together with your doctor to make sure your medications are the best for you. |  |  |  |  |  |
| 23 | The responsibility that the pharmacist assumes for your drug therapy |  |  |  |  |  |
| **General Satisfaction** | | | | | |  |
| 24 | The privacy of conversations with the pharmacist |  |  |  |  |  |
| 25 | The amount of time it takes to get a prescription filled at your pharmacy |  |  |  |  |  |
| 26 | The professional appearance of the pharmacy |  |  |  |  |  |
| 27 | Pharmacy’s services overall |  |  |  |  |  |
| **Other satisfaction parameters** | | | | | |  |
| **No** | **Question** | **Agree** | | **Disagree** | |  |
| 28 | The location of the pharmacy was convenient and comfortable |  | |  | |  |
| 29 | Medication quantity was sufficient |  | |  | |  |
| 33 | The waiting area was comfortable |  | |  | |  |
| 31 | The pharmacy area was clean and acceptable |  | |  | |  |
| 32 | All medications were available in the pharmacy |  | |  | |  |
| 33 | All medications I received were well packaged |  | |  | |  |
